# Comparative analysis of CRASH and IMPACT in predicting the outcome of 340 patients with traumatic brain injury

**DOI:** 10.1515/tnsci-2022-0327

**Published:** 2024-03-22

**Authors:** Tingting An, Zibei Dong, Xiangyang Li, Yifan Ma, Jie Jin, Liqing Li, Lanjuan Xu

**Affiliations:** Department of Critical Care Medicine, Zhengzhou Central Hospital affiliated to Zhengzhou University, Zhengzhou, Henan, 450001, China

**Keywords:** traumatic brain injury, prediction model: CRASH, IMPACT

## Abstract

**Background:**

Both the International Mission for Prognosis and Analysis of Clinical Trials (IMPACT) and the Corticosteroid randomization after significant head injury (CRASH) models are globally acknowledged prognostic algorithms for assessing traumatic brain injury (TBI) outcomes. The aim of this study is to externalize the validation process and juxtapose the prognostic accuracy of the CRASH and IMPACT models in moderate-to-severe TBI patients in the Chinese population.

**Methods:**

We conducted a retrospective study encompassing a cohort of 340 adult TBI patients (aged > 18 years), presenting with Glasgow Coma Scale (GCS) scores ranging from 3 to 12. The data were accrued over 2 years (2020–2022). The primary endpoints were 14-day mortality rates and 6-month Glasgow Outcome Scale (GOS) scores. Analytical metrics, including the area under the receiver operating characteristic curve for discrimination and the Brier score for predictive precision were employed to quantitatively evaluate the model performance.

**Results:**

Mortality rates at the 14-day and 6-month intervals, as well as the 6-month unfavorable GOS outcomes, were established to be 22.06, 40.29, and 65.59%, respectively. The IMPACT models had area under the curves (AUCs) of 0.873, 0.912, and 0.927 for the 6-month unfavorable GOS outcomes, with respective Brier scores of 0.14, 0.12, and 0.11. On the other hand, the AUCs associated with the six-month mortality were 0.883, 0.909, and 0.912, and the corresponding Brier scores were 0.15, 0.14, and 0.13, respectively. The CRASH models exhibited AUCs of 0.862 and 0.878 for the 6-month adverse outcomes, with uniform Brier scores of 0.18. The 14-day mortality rates had AUCs of 0.867 and 0.87, and corresponding Brier scores of 0.21 and 0.22, respectively.

**Conclusion:**

Both the CRASH and IMPACT algorithms offer reliable prognostic estimations for patients suffering from craniocerebral injuries. However, compared to the CRASH model, the IMPACT model has superior predictive accuracy, albeit at the cost of increased computational intricacy.

## 1 Introduction

Traumatic brain injury (TBI) is the leading cause of mortality and morbidity among younger individuals globally, with substantial variations in etiological factors, pathological manifestations, severity levels, and prognostic outcomes [1]. Prognostic models, which combine various patient-specific characteristics, are critical for facilitating early clinical decision-making, designing tailored patient management strategies, informing research paradigms, and interpreting outcomes in clinical trials [2]. The International Mission for Prognosis and Analysis of Clinical Trials (IMPACT) [3] and the Corticosteroid randomization after significant head injury (CRASH) [4] are currently the two principal prognostic algorithms that are extensively deployed to compute the mortality and prognostic outcomes of TBI patients across large datasets.

Both models synergistically integrate clinical indices, computed tomography (CT) results, and laboratory markers at admission to prognosticate the 14-day mortality risk and the 6-month outcomes. Although these models have undergone a myriad of external validations globally since inception, continuous external validations are still required to further substantiate their universal applicability across diverse healthcare ecosystems. A 2020 seminal study encompassing 13,627 TBI patients across 56 centers in 22 Chinese provinces revealed inter-center and regional discrepancies in mortality rates. Specifically, the CRASH basic model predicted a 14-day mortality rate of 1,116 (13%), which differed considerably from the observed 14-day mortality rate of 544 (7%); the odds ratio of observation to expectation was documented at 0.49 [95% CI: 0.45–0.53] [5]. Another study analyzed the data of 635 patients out of 1,091 patients registered in the Japan Neurotrauma Data Bank (JNTDB). Specifically, they examined factors associated with in-hospital mortality and unfavorable outcomes at 6 months post-TBI by applying the TRISS, CRASH, and IMPACT models. Furthermore, they externally validated these models based on the available data. According to the results, the CRASH (basic and CT) and IMPACT (core and core extended) models had satisfactory area under the curve (AUC) values for unfavorable outcomes at 6 months (0.86, 0.86, 0.81, and 0.85, respectively). The CRASH and IMPACT models were applicable to the JNTDB population, indicating their high value in Japanese neurotrauma patients [6].

Herein, we aim to externally validate both the IMPACT and CRASH prognostic algorithms using a patient cohort from our institution to comparatively evaluate their capacities to predict mortality and unfavorable outcomes. Our findings may offer valuable insights that could potentially inform and refine management decisions within the field of TBI care.

## 2 Object and methods

### 2.1 Research object

The current survey is a retrospective cohort study which was conducted at the Zhengzhou Central Affiliated Hospital, Zhengzhou University. The research sample included TBI patients admitted to the hospital between 2020 and 2022. The research hospital has an Intensive care unit (ICU) equipped with modern monitoring and rescue facilities, as well as intensive care specialists and all-weather neurosurgery services to maximize patients’ survival and subsequent Quality of life (QoL), and can provide 24 h intensive treatment and nursing. All patients were managed as per the latest TBI treatment guidelines [7].

The inclusion criteria were as follows: (1) Patients aged ≥ 18 years old; (2) Patients diagnosed with moderate to severe TBI (Glasgow Coma Scale; GCS is 3–12) through imaging (CT or MRI); and (3) Patients admitted within 12 h of TBI.

The exclusion criteria were as follows: (1) Patients with previous history of nervous system trauma; (2) Patients with penetrating craniocerebral injury; (3) Expectant women; (4) Patients participating in other clinical studies at the time of the survey; and (5) Patients with incomplete data (patients lacking key predictive indicators such as GCS scores and CT examination).

### 2.2 IMPACT and CRASH models

The IMPACT model has three major components: The core model (age, motor score, and pupil response to light); the CT model (hypoxia, hypotension, CT rating, traumatic subarachnoid hemorrhage, and epidural hematoma, which are all added to the basic model); and the Lab model (based on the CT model, and the blood sugar and hemoglobin concentrations). Each model component can calculate two kinds of results using an online website (available at: http://www.tbi-impact.org/?p=impact/calc): 6-month mortality and 6-month unfavorable Glasgow Outcome Scale (GOS) scores. On the other hand, the CRASH model has two major components: The basic model (age, GCS, pupil reactivity, and whether there is major extracranial injury) and the CT model (incorporates the results of the first CT scan post-injury). Each component model can calculate two kinds of results using an online website (available at: http://www.crash.lshtm.ac.uk/Risk%20calculator/index.html): 14-day mortality and 6-month unfavorable GOS scores.

### 2.3 Data collection

Data were meticulously extracted from the electronic medical record system. Variables analyzed included demographic factors (age, sex, and so on), clinical and physiological indicators (GCS score and vital signs at admission), trauma severity metrics (Injury Severity Score; ISS and Abbreviated Injury Score; AIS), etiological elements (cause of injury), and temporal markers (time interval from injury to admission, ICU stay, and duration of hospitalization). Data pertaining to extracranial major injuries, pupillary reactivity, emergent surgical treatments, physical activity scores, and markers of oxygenation (hypoxia), perfusion (hypotension), and hemoglobin levels at admission were also scrutinized.

### 2.4 Statistical analysis

Statistical analyses were performed using the SPSS 22.0 software package. Quantitative data were presented as mean value (M) ± standard deviation (SD) or as interquartile ranges (IQRs), depending on their distributional properties. Qualitative or categorical data were expressed as frequency counts and percentages. The analytical rigor of the prognostic models was assessed by computing the area under the receiver operating characteristic curve (AUROCC) within a 95% confidence interval (CI) to quantify the models’ discriminatory capabilities. Models with AUC values of <0.5, >0.7, and >0.8 were considered non-discriminant, sufficiently discriminant, and exceptionally discriminant, respectively. The Brier score was used to evaluate the models’ calibration degree. The range of the Brier score was 0∼1. The closer the Brier score is to 0, the better the model’s calibration degree. The model has no predictive ability when the Brier score equals 1. The total GCS was collected and availed for imputation and analysis in cases where specific data on eye opening, verbal response, and motor response were missing. Missing data among the predictors were addressed through the multiple imputation approach.


**Ethical approval:** The research related to human use has been complied with all the relevant national regulations, institutional policies and in accordance the tenets of the Helsinki Declaration, and has been approved by the authors’ institutional review board or equivalent committee. Studies involving human participants were reviewed and approved by the Ethics Committee of Zhengzhou Central Hospital (No.202263).
**Informed consent:** Informed consent has been obtained from all individuals included in this study. The patients’ right to informed consent was waived for this study in accordance with national legislation and institutional requirements.

## 3 General data analysis

Herein, 340 TBI patients (average age = 54 ± 17.4 years, with male predominance of 69.5% [*n* = 146]) were included. The median GCS score upon admission was 8. Concomitant major extracranial trauma, which was defined as a single AIS > 3, was found in 150 patients (44.12%). Hypoxic conditions and hypotension (blood pressure < 90/60 mmHg) were observed in 83 (24.27%) and 52 (15.21%) patients, respectively. Subarachnoid hemorrhage was a common pathological feature, occurring in 306 patients (89.47%). On the other hand, loss of pupillary light reflex was documented in 72 patients (21.18%). At admission, the median hemoglobin and blood glucose levels were 12 g/dL and 7.9 mmol/L, respectively. Longitudinal follow-up revealed that 223 patients (65.59%) had an unfavorable prognosis (GOS score < 4). The observed mortality rate was 40.29% (137), with 22.06% (75) succumbing within the first 14 days. Persistent vegetative states and severe disabilities were observed in 38 (11.18%) and 48 (14.12%) patients, respectively.

Table 1 summarizes and compares the participant characteristics with the IMPACT and CRASH datasets. According to the Marshall score for CT findings, patients included herein had more severe brain pathology. The GCS and Motor scores revealed that patients’ responses in this study were also poor. Furthermore, the proportion of the six-month GOS unfavorable outcomes in this study was slightly higher (65.59%).

**Table 1:** Patient characteristics as compared against the IMPACT and CRASH datasets

Variables	This study	IMPACT	CRASH
	*N* = 340	*n* = 8,509	*n* = 6,681
Inclusion period	2020–2022	1984–1997	1999–2004
Gender (male)	243 (71.47)	—	—
Age (years), median (IQR)	54.85 (38–65)	30 (21–45)	32 (23–47)
Time from injury to admission (h), median (IQR)	1 (1–2)	—	—
GCS post-resuscitation			
Severe (3–8)	77.5%	82%	39.5%
Median (IQR)	7 (4–11)	—	—
ISS, median (IQR)	26 (20–33)	—	—
AIS of the head, median (IQR)	4 (4–5)	—	—
Major extracranial injury, *n* (%)	150 (44.12%)	—	1,735 (27%)
Hypoxia, *n* (%)	83 (24.27%)	1,116 (20%)	—
Hypotension, *n* (%)	52 (15.21%)	1,171 (18%)	—
Subarachnoid hemorrhage, *n* (%)	306 (89.47%)	3,313 (45%)	2,045 (36%)
Epidural hematoma, *n* (%)	121 (35.38%)	999 (13%)	—
Marshall CT classification, *n* (%)			
Diffuse injury I	19 (5.59%)	360 (7%)	954 (17%)
Diffuse injury II	98 (28.82%)	1,838 (35%)	1,517 (27%)
Diffuse injury III	125 (36.77%)	863 (17%)	604 (11%)
Diffuse injury IV	35 (10.29%)	187 (4%)	133 (2%)
Evacuated mass lesion (V) and non- evacuated mass lesion (VI)	63 (18.53%)	1,944 (38%)	2,446 (43%)
Pupils, *n* (%)			
Both reactive	186 (54.71%)	—	—
One or both pupils are nonreactive	121 (35.59%)	2,640 (37%)	1,316 (20%)
Motor score, *n* (%)			
Without reactivity	72 (21.18%)	1,395 (16%)	785 (12%)
Hyperextension	40 (11.77%)	1,042 (12%)	515 (8%)
Abnormal flexion	69 (20.29%)	1,085 (13%)	658 (10%)
Normal flexion	80 (23.53%)	1,940 (23%)	1,156 (17%)
Obeys	65 (19.12%)	2,591 (30%)	3,567 (53%)
Untestable or missing	15 (4.41%)	456 (5%)	0
Hb (g/dL), median (IQR)	12 (11–14)	13 (11–14)	—
Glucose level (mmol/L), median (IQR)	7.9 (6.5–9.6%)	8.2 (6.7–10.4)	—
14-day death, *n* (%)	75 (22.06%)	—	1,902 (19.5%
6-month GOS outcome, *n* (%)			
Death	137 (40.29%)	2,396 (28%)	2,146 (32%)
Vegetative state	38 (11.18%)	351 (4%)	993 (15%)
Severe disability	48 (14.12%)	1,335 (16%)	171 (12.1%)
Moderate disability	52 (15.29%)	1,666 (20%)	1,224 (18%)
Favorable recovery	65 (19.12%)	2,761 (32%)	2,318 (35%)
6-month GOS unfavorable outcome, n (%)	223 (65.59%)	4,082 (48%)	3,310 (59.1%)
6-month death, *n* (%)	137 (40.29%)	2,396 (28%)	2,146 (32%)

### 3.1 Performance of the IMPACT and CRASH models

#### 3.1.1 IMPACT model

**Figure 1:**
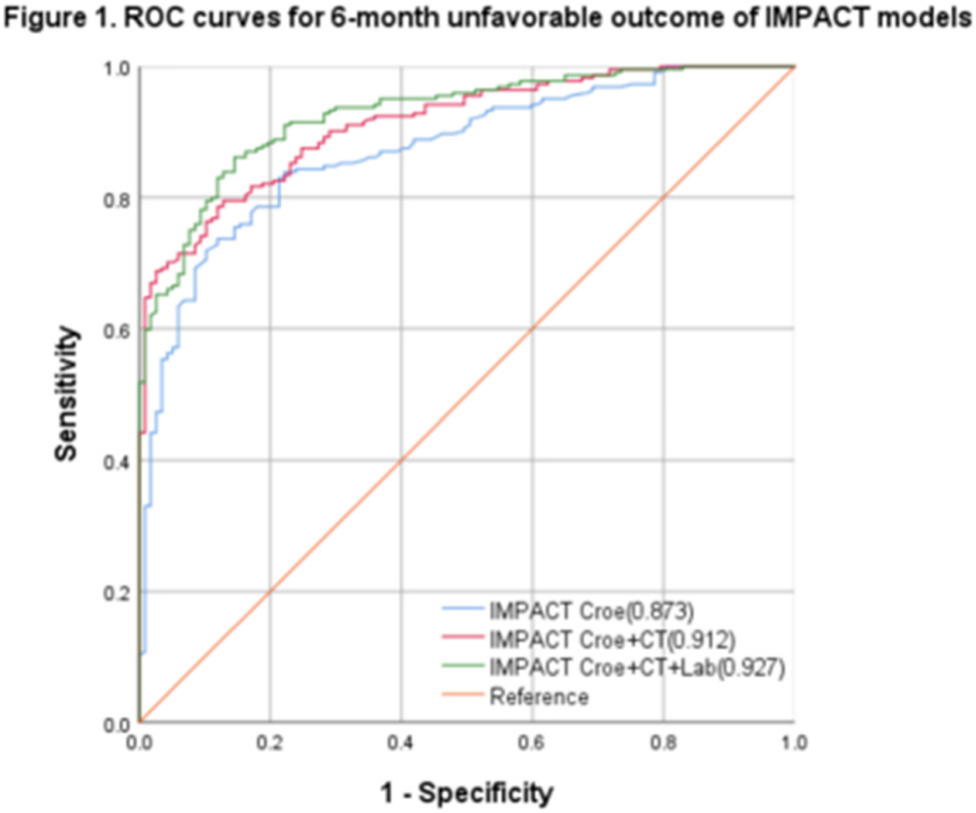
The IMPACT model’s ROC curves for the 6-month unfavorable outcomes.

**Figure 2:**
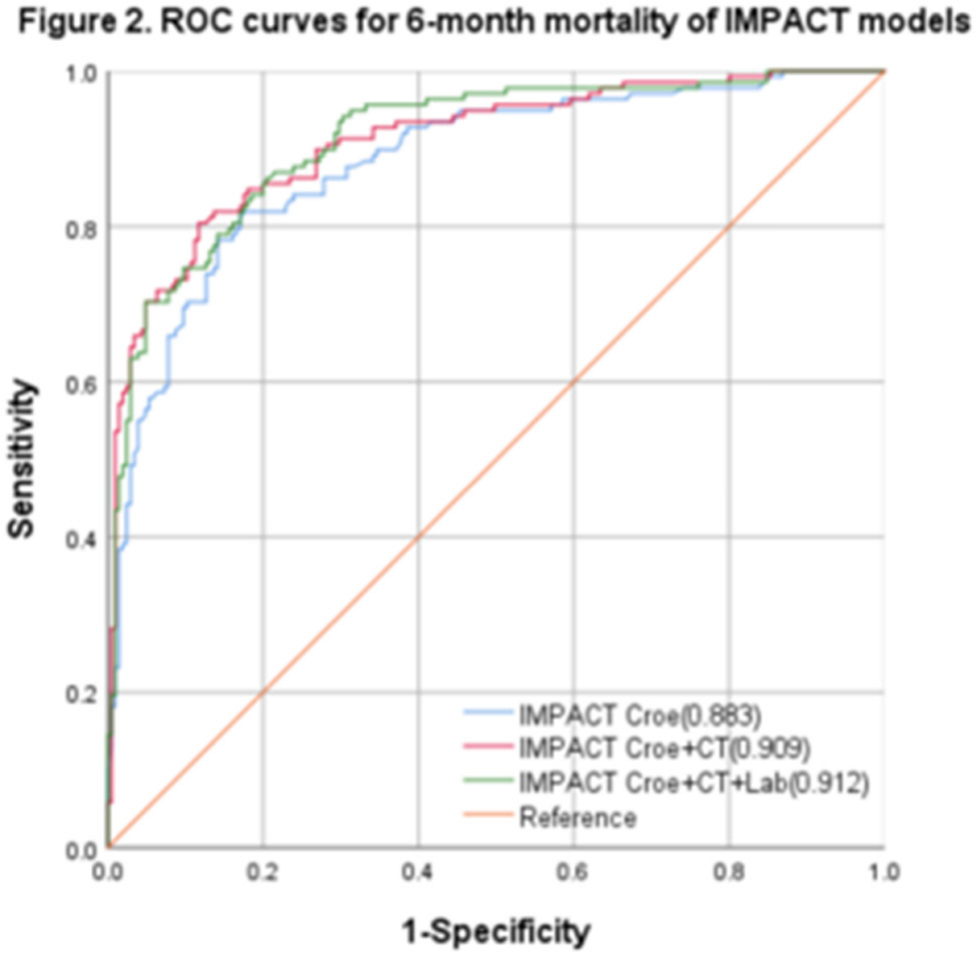
The IMPACT model’s ROC curves for the 6-month mortality.

The IMPACT model’s ROC curve was generated in relation to diverse clinical outcomes, and the AUC was calculated (Figures 1 and 2). The AUC values for the 6-month unfavorable TBI outcomes were found to be 0.873 (95% CI: 0.835–0.910, *P* < 0.001), 0.912 (95% CI: 0.884–0.941, *P* < 0.001), and 0.927 (95% CI: 0.900–0.953, *P* < 0.001). The corresponding best cut-off values were 0.621, 0.670, and 0.720, respectively. The corresponding sensitivity values were 0.843, 0.798, and 0.865, respectively. On the other hand, the corresponding specificity values were 0.778, 0.872, and 0.855, respectively. Finally, the corresponding Brier scores were 0.14, 0.12, and 0.11, respectively. The IMPACT model’s AUC values for the 6-month mortality prediction were 0.883 (95% CI: 0.847–0.921, *P* < 0.001), 0.909 (95% CI: 0.879–0.943, *P* < 0.001), and 0.912 (95% CI: 0.882–0.944, *P* < 0.001). The corresponding best cut-off values were 0.648, 0.692, and 0.661, respectively. The corresponding sensitivity values were 0.852, 0.810, and 0.847, respectively. The corresponding specificity values were 0.823, 0.882, and 0.814, respectively. The Brier scores were 0.15, 0.14, and 0.13, respectively (Table 2).

**Table 2:** IMPACT model’s verification

Models		AUC	95%CI	*P*	Best cut-off	Sensitivity	Specificity	Brier score
IMPACT Core	6-month unfavorable outcome	0.873	0.835–0.910	＜0.001	0.621	0.843	0.778	0.14
	6-month mortality	0.883	0.847–0.921	＜0.001	0.648	0.852	0.823	0.15
IMPACT Core + CT	6-month unfavorable outcome	0.912	0.884–0.941	＜0.001	0.670	0.798	0.872	0.12
	6-month mortality	0.909	0.879–0.943	＜0.001	0.692	0.810	0.882	0.14
IMPACT Core + CT + Lab	6-month unfavorable outcome	0.927	0.900–0.953	＜0.001	0.720	0.865	0.855	0.11
	6-month mortality	0.912	0.882–0.944	＜0.001	0.661	0.847	0.814	0.13

#### 3.1.2 CRASH model

**Figure 3:**
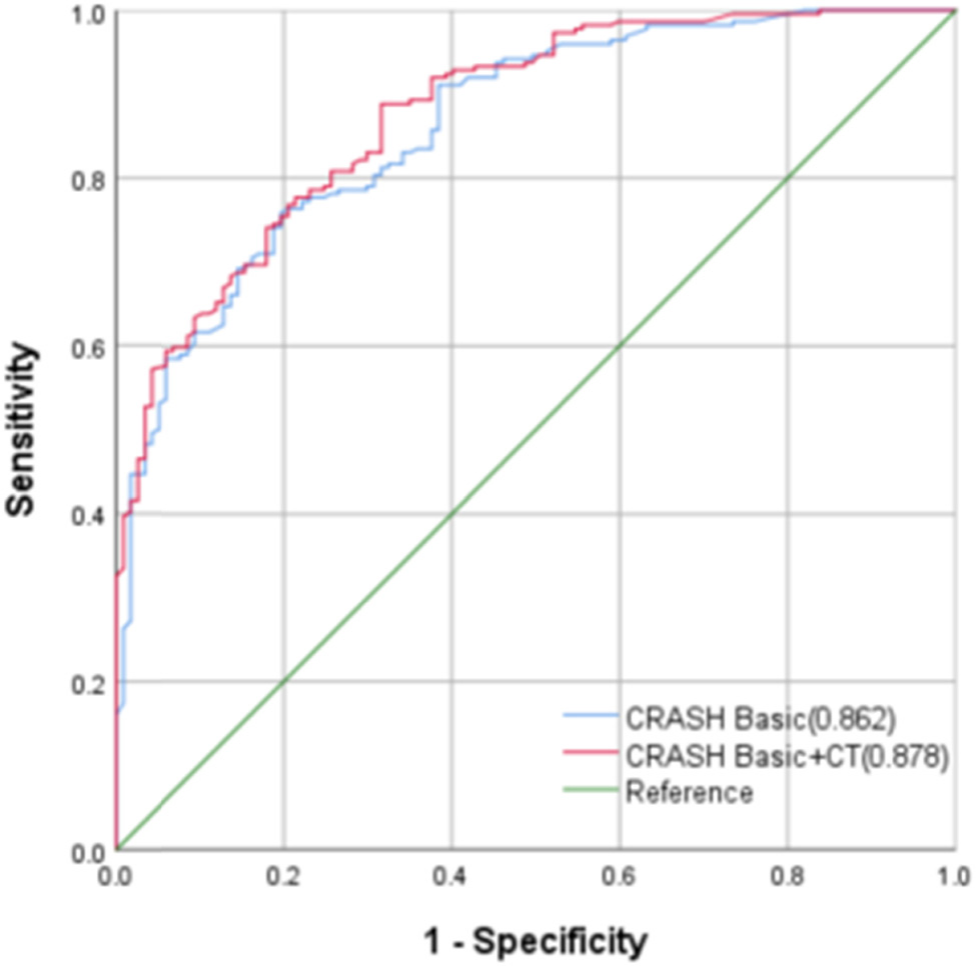
The CRASH model’s ROC curves for the 6-month unfavorable outcomes.

**Figure 4:**
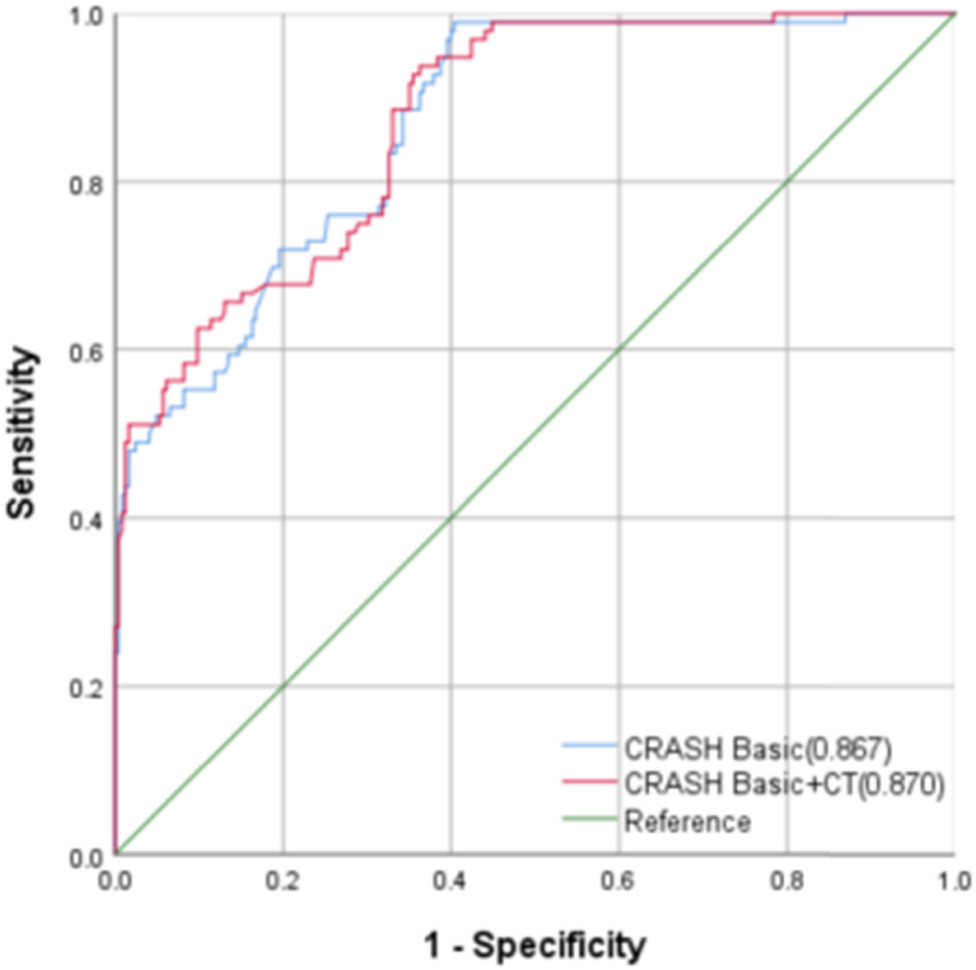
The CRASH model’s verification.

The CRASH model’s ROC curves were generated in relation to diverse clinical outcomes, and the AUC was calculated (Figures 3 and 4). The AUC values for the 6-month mortality prediction were found to be 0.862 (95% CI: 0.823–0.901, *P* < 0.001) and 0.878 (95% CI: 0.842–0.914, *P* < 0.001). The corresponding best cut-off values were 0.561 and 0.585, respectively. The corresponding sensitivity values were 0.756 and 0.990, respectively. The corresponding specificity values were 0.805 and 0.595, respectively. The Brier scores were both 0.18. The AUC values for the CRASH model’s 14-day mortality predictions were 0.867 (95% CI: 0.827–0.906, *P* < 0.001) and 0.870 (95% CI: 0.831–0.91, *P* < 0.001). The corresponding best cut-off values were 0.572 and 0.575, respectively. The corresponding sensitivity values were 0.888 and 0.938, respectively. Finally, the corresponding specificity values were 0.684 and 0.637, respectively (Table 3).

**Table 3:** ROC curve analysis for CRASH models

Models		AUC	95% CI	*P*	Best cut-off	Sensitivity	Specificity	Brier score
CRASH Basic	6-month unfavorable outcome	0.862	0.823–0.901	＜0.001	0.561	0.756	0.805	0.18
	14-day mortality	0.867	0.827–0.906	＜0.001	0.572	0.888	0.684	0.21
CRASH Basic + CT	6-month unfavorable outcome	0.878	0.842–0.914	＜0.001	0.585	0.990	0.595	0.18
	14-day mortality	0.870	0.831–0.910	＜0.001	0.575	0.938	0.637	0.22

## 4 Discussion

Navigating the intricate landscape of TBI management poses a formidable challenge, primarily because of the substantial heterogeneity in both the pathological manifestation and prognostic outcomes of TBI [8,9]. Ascertaining reliable predictive outcomes is critical for clinicians, patients, and their families alike [10]. Herein, the external validity of the IMPACT and CRASH prognostic models were exhaustively evaluated using 340 TBI patients admitted to our institution between 2020 and 2022. Both models exhibited commendable discriminatory capabilities. Furthermore, a nuanced observation revealed that the models’ complexity was positively correlated with their discriminatory capabilities. Based on the AUC metrics, the IMPACT Lab model was the most effective, especially in forecasting the 6-month mortality and adverse outcomes in this particular cohort (AUC values of 0.892 and 0.845, respectively). This can be attributed to the Lab model’s comprehensive nature, which incorporates CT-captured variables indicative of secondary injuries and intracranial aberrations, as well as variables signifying potential pathophysiological processes. The model’s predictive acumen amplifies the synergistic effect of these variables.

Over the past decade, both the IMPACT and CRASH models have been subjected to various external validations across high-income [11,12,13] as well as low-to-middle-income nations [14,15,16]. However, more developmental iterations and validation procedures are required to ascertain their universal applicability across diverse healthcare settings. A contemporaneous systematic review focusing on prognostic models for moderate to severe TBI demonstrated that both the IMPACT and CRASH models maintained a moderate to good discriminatory capability in diverse settings, with an average AUC ranging from 0.77 to 0.82 [10]. These findings were corroborated by a large-cohort European study involving 1,742 patients, which established that both models exhibited robust discriminatory features, with AUC values of 0.80–0.88 and 0.82–0.88 for the IMPACT and CRASH models, respectively, even though calibration was generally moderate [17]. Contrastingly, a specialized neurosurgical study at the University of Pittsburgh, which prospectively assessed severe TBI cases in a single Level I trauma center (*n* = 467), discovered that although both models demonstrated good discrimination (AUC = 0.77–0.81), they exhibited a somewhat optimal overall performance in mortality and poor prognosis prediction [18]. Furthermore, a large study leveraging the national trauma database encompassing 26,228 patients affirmed that both models could discriminate between survival and mortality outcomes. However, compared to the CRASH model (0.858; 95% CI: 0.854–0.863), the IMPACT model had a marginally superior AUC (0.863; 95% CI: 0.858–0.867), while manifesting a slight calibration discrepancy; specifically, it over-predicted and under-predicted at lower and higher scores, respectively [19].

Our results revealed that the optimal cut-off values for predicting the 6-month adverse outcomes in TBI patients using the CRASH Basic and CRASH Basic + CT models were 0.561 and 0.585, respectively. These models had corresponding sensitivities of 75.6 and 99%, while their specificities were limited to 80.5 and 59.5%. Such specificities imply that only 80.5 and 59.5% of patients are likely to experience a favorable prognosis when predicted risk is below these delineated thresholds, resulting in a substantial 19.5 and 40.5% margin of error, thereby calling into question the CRASH model’s practical applicability in clinical settings. Conversely, the IMPACT Core model demonstrated an optimal cut-off value of 62.1, with corresponding sensitivity and specificity rates of 84.3 and 77.8%, respectively. These elevated metrics highlight the superior clinical applicability and predictive precision of the IMPACT Core model. The Brier score for the IMPACT model ranged between 0.11 and 0.15 (significantly lower than that of the CRASH model), further validating its efficacy. These findings collectively suggest that the IMPACT model outperformed the CRASH model in accurately forecasting the 6-month adverse outcomes in TBI patients, at least within the confines of this specific dataset. Furthermore, we observed that although the core clinical predictors (age, GCS score, and pupillary reactivity) were crucial in comprehensively identifying TBI patients with high mortality or poor prognosis, the correction results of the core model were poor compared to the more complex models, highlighting the need to adjust the model to suit the specific clinical environment [20].

Various research characteristics influence the discriminative capabilities of these models during external validation [21]. Notably, case-mix divergence between the validation and development cohorts can significantly impact the models’ performance. For instance, the IMPACT model’s validation cohort predominantly comprised patients with severe TBI (72% of cases). The cohort registered a mortality rate of 30% at the 6-month mark, with 55% of the patients experiencing poor prognoses [19]. On the other hand, the CRASH model’s cohort comprised patients with a broader spectrum of TBI severity, with mild cases constituting 30% of the included patients. Furthermore, the majority of the CRASH cohort patients (75%) were from low-income countries, with a comparatively simplified data collection protocol [4]. Herein, the observed mortality rate exceeded the rate predicted by the IMPACT model, and a greater number of adverse outcomes was recorded. This elevated mortality could be attributed to the low GCS scores observed at admission and the specific demographic characteristics of the patient cohort. Furthermore, racial and ethnic factors may have influenced the observed mortality rates.

In summary, the external verification of the IMPACT and CRASH models in this study proves their predictive value for the Chinese TBI population. Based on the favorable discriminatory abilities of the IMPACT and CRASH models, regularly comparing these models’ prognostic results with clinical expectations might help clinicians adjust their predictions and practices [22–24]. Moreover, decisions on which model to use should be primarily based on specific environmental or population characteristics (such as TBI severity and the economic situation of a country). Additionally, the use of IMPACT and CRASH models and their complexity depend on the availability of predictive parameters.

However, it is imperative to acknowledge that the study’s single-institutional design is a pivotal limitation. The clinical practices, sample size, and expertise of healthcare providers at this singular institution could introduce an element of bias into the outcomes, necessitating additional research with diverse datasets for more robust external validation. Furthermore, the integration of multiparametric predictors presents a promising avenue for refining these prognostic models. Recent scientific endeavors have begun to incorporate more variables, such as coagulation factors [25], biomarkers [26], and other clinically relevant indicators into new prognostic models. Such advancements aim to enhance the precision of outcome predictions post-TBI. This multi-variate prognostic approach constitutes an area of continued interest and will be a focal point in our research team’s subsequent investigations.
